# Crowding-Activity Coupling Effect on Conformational Change of a Semi-Flexible Polymer

**DOI:** 10.3390/polym11061021

**Published:** 2019-06-10

**Authors:** Xiuli Cao, Bingjie Zhang, Nanrong Zhao

**Affiliations:** College of Chemistry, Sichuan University, Chengdu 610064, China; caoxiuli54@126.com (X.C.); zbjscu2016@163.com (B.Z.)

**Keywords:** polymer conformational change, crowding effect, activity effect, Langevin simulation

## Abstract

The behavior of a polymer in a passive crowded medium or in a very dilute active bath has been well studied, while a polymer immersed in an environment featured by both crowding and activity remains an open problem. In this paper, a systematic Langevin simulation is performed to investigate the conformational change of a semi-flexible chain in a concentrated solution packed with spherical active crowders. A very novel shrinkage-to-swelling transition is observed for a polymer with small rigidity. The underlying phase diagram is constructed in the parameter space of active force and crowder size. Moreover, the variation of the polymer gyration radius demonstrates a non-monotonic dependence on the dynamical persistence length of the active particle. Lastly, the activity-crowding coupling effect in different crowder size baths is clarified. In the case of small crowders, activity strengthens the crowding-induced shrinkage to the chain. As crowder size increases, activity turns out to be a contrasting factor to crowding, resulting in a competitive shrinkage and swelling. In the large size situation, the swelling effect arising from activity eventually becomes dominant. The present study provides a deeper understanding of the unusual behavior of a semi-flexible polymer in an active and crowded medium, associated with the nontrivial activity-crowding coupling and the cooperative crowder size effect.

## 1. Introduction

Active particles, also known as self-propelled Brownian particles or microswimmers, can utilize external or internal energy for direct motion and put various processes out of equilibrium [1,2,3,4,5]. In the biological realm, active matter ranges from the cytoskeleton in living cells to algae, sperm, and bacteria cells [6,7,8,9,10,11,12,13]. Activity allows microorganisms a more efficient exploration of the environment and also makes microswimmers exhibit a series of novel properties and collective dynamics [14,15,16]. In recent years, growing efforts have been devoted to obtaining a deeper understanding of the unique behaviors of active systems. These studies definitely promote hopeful applications in a wide range of disciplines including statistical physics [1], fluid mechanics [17,18], biology [19], biomedicine [20], and so on.

In general, crowding is another significant property of bio-related active systems [21]. For instance, biomolecules within cytoplasm in a living cell are coupled with an active surrounding due to the presence of molecular motors [22,23]. Simultaneously, the cytoplasm is also a typical crowded environment, since it is generally occupied by a number of proteins, DNA, and ribosomes, up to about 30% volume fraction [24]. Cytoplasmic crowding in fact leads to striking phenomena, such as the emergence of anomalous diffusion [25,26,27]. Furthermore, crowding plays a central role in the structural and dynamical properties of biological macromolecules, thus modulating the relevant rate processes, including structural transitions of disordered protein [28,29], RNA folding [30,31], DNA looping [32,33], genome compaction [34], etc.

Up to date, conformational changes of biopolymers in complex media with either the activity or crowding feature have gained much attention. On the one hand, several experiments have revealed that active processes greatly affect the polymers’ and motor proteins’ shapes and functions [7,35]. Theoretically, Langevin simulation has been widely used to evaluate the structural and dynamical behaviors of a polymer in a well-defined thermal bath. Kaiser et al. [36] investigated the conformation and dynamics of the self-avoiding flexible polymer chain in an active bacterial bath. An unusual swelling and a crossover to the universal Flory scaling behavior were observed. Similarly, Harder et al. [37] studied the elastic properties of a rigid filament in a bath of self-propelled particles. They showed that activity could induce collapse and re-expansion upon increasing the strength of propelling force. These pioneering studies were mainly dedicated to unraveling the activity effect by considering very dilute active baths, where the crowding property has been effectively excluded. On the other hand, there are plenty of studies focusing on the crowding effect. Experiments showed that macromolecular crowding modestly reduced the size of intrinsically-disordered proteins [38]. In addition, numerical simulations observed an evident collapse effect to a biopolymer induced by macromolecular crowding. The degree of shrinkage was found to vary from system to system, depending on crowder shape, concentration, and crowder size in rather complicated manners [39]. These studies concerning the crowding effect so far have been mostly limited to a passive bath, which surely excludes the complexity of activity coupling. As mentioned, a realistic biological environment possibly involves the coexistence of both crowding and activity, that is the crowding agents are self-propelled. Under such situations, a systematic study that properly takes into account the coupling between crowding and activity should be mandatory. Unfortunately, to the best of our knowledge, a comprehensive understanding of such an issue is still lacking.

Furthermore, crowder size should be one of the important factors determining how the crowding and activity exert their roles on the above problem. The size effect associated with crowding-induced collapse in passive baths has been well analyzed. For example, simulations for a bead-spring chain in a solution crowded by passive hard sphere particles showed that the degree of collapse increased as the size of crowder decreased, and a coil-globe transition of probed polymer could be possibly induced with sufficiently small crowders [39]. In active matter system, most studies rationalized the degree of activity effect in terms of active force rather than crowder size. It is worth noting that the active particle is characterized by a dynamical persistence length *L*, which measures the distance of the particle moving along the direction of its original orientation. As is known, supposing a particle with diameter $\sigma_{a}$ is subject to an active force $F_{a}$, its dynamical persistence length is given by $L=v\tau_{r}=F_{a}\sigma_{a}^{2}/3k_{B}T$ [5]. $\tau_{r}$ is the characteristic time scale given by the inverse of the particle’s rotational diffusion coefficient $D_{r}=\tau_{r}^{-1}=\frac{k_{B}T}{\pi \eta_{0}\sigma_{a}^{3}}$, where $k_{B}$ is the Boltzmann constant, *T* is the absolute temperature, and $\eta_{0}$ is the fluid viscosity. $v=F_{a}/\gamma_{a}$ denotes the self-propulsion speed along its original orientation, with $\gamma_{a}=3\pi \eta_{0}\sigma_{a}$ being the particle’s Stokes friction coefficient. It is evident that both active force $F_{a}$ and active particle size $\sigma_{a}$ play similar important roles to maintain the particle’s self-propulsion and activity features. The size effect is definitely a key factor, in particular in an active-crowded medium, and results in rather complicated consequences to the relevant problems.

Motivated by the above considerations, in the present work, we investigate the conformational change of a semiflexible polymer in an active and crowded bath based on coarse-grained Langevin simulation. We extensively analyze the dependence of polymer gyration radius on chain rigidity, bath volume fraction, the strength of active force, as well as the crowder size. In addition, the associated shape parameter of the chain, the effective temperature, and the gyration radius distribution will be examined. Particular attention is paid to revealing the crucial activity-crowding coupling effect. More importantly, as a comparative study, the cooperative crowder size effect will be also specifically unraveled.

This paper is organized as follows: Section 2 defines the simulation model and method. In Section 3, we present our main results and discussion. We firstly address the activity effect under varying crowder sizes. Then, we clarify the activity-crowding coupling effect. Finally, we conclude our paper in Section 4.

## 2. Model and Method

We studied the conformational change of a semi-flexible polymer in an active bath based on a two-dimensional Langevin dynamical simulation. The bath was crowded by active particles, with a certain value of volume fraction ϕ, such that the crowding-coupling effect can be involved in our model.

The probed polymer was modeled as a bead spring chain consisting of *N* coarse-grained monomers of diameter $\sigma_{b}$, linearly connected via harmonic bonds according to

(1) $$U_{bond}=\frac{k_{b}}{2}\sum_{i=2}^{N}\left(\right|r_{i}-r_{i-1}{\left|-l_{0}\right)}^{2}$$

where $k_{b}$ is the spring constant. $|r_{i}-r_{i-1}|$ refers to the center-to-center distance between linked monomers *i* and i−1. $l_{0}=\sigma_{b}$ is the equilibrium bond length.

An additional angular interaction energy was used to introduce rigidity to the polymer:

(2) $$U_{angle}=\kappa {\left(\phi_{angle}-\pi \right)}^{2}$$

where κ is the interaction strength parameter measuring the degree of rigidity and $\phi_{angle}$ denotes the angle between adjacent bonds.

We assumed the non-bond interactions between any two sites *i* and *j* in the system, including the chain monomers and the active particles, were modeled by the purely repulsive Weeks–Chandler–Andersen (WCA) potential:

(3) $$U_{non-bond}\left(r_{ij}\right)=\left\{\begin{array}{c} \epsilon \left[{\left(\frac{\sigma_{ij}}{r_{ij}}\right)}^{12}-{\left(\frac{\sigma_{ij}}{r_{ij}}\right)}^{6}+\frac{1}{4}\right],\;\;\;\;\;r_{ij}\leq r_{cut}\\ 0,\;r_{ij}>r_{cut} \end{array}\right.$$

where $\sigma_{ij}=\left(\sigma_{i}+\sigma_{j}\right)/2$, with $\sigma_{i}$, $\sigma_{j}$ denoting the diameter of particle sites *i* and *j*, respectively. The energy is truncated at $r_{cut}=2^{1/6}\sigma_{ij}$. ϵ, and $r_{ij}$ denotes the interaction strength and the site-to-site distance.

The dynamics of the *i*^th^ chain monomer was described by the Langevin equation:

(4) $$m_{b}\frac{d^{2}r_{i}}{dt^{2}}=-\gamma_{b}\frac{dr_{i}}{dt}-\nabla_{r_{i}}\left(U_{bond}+U_{angle}+U_{non-bond}\right)+\Gamma_{i}\left(t\right)$$

Here, $r_{i}$ denotes the position of the monomer, $m_{b}$ being the bead mass. $\gamma_{b}$ is the friction coefficient of the monomer in the background of pure solvent. $\Gamma_{i}\left(t\right)$ is independent Gaussian white noise with zero mean and autocorrelation functions $\langle \Gamma_{i}\left(t\right)\Gamma_{j}\left(t^{\prime }\right)\rangle =2k_{B}T\gamma_{b}I\delta_{ij}\delta \left(t-t^{\prime }\right)$, where I is the unit matrix and $\delta_{ij}$ is the Kronecker delta symbol.

For the active particle, the direction of motion is subject to rotational diffusion, which leads to a coupling between rotation and translation. The stochastic differential equation to describe the position of each active particle $r_{a}$ is given by [36,37,40,41]:

(5) $$\begin{array}{c} m_{a}\frac{d^{2}r_{a}}{dt^{2}}=-\gamma_{a}\frac{dr_{a}}{dt}-\nabla_{r_{a}}U_{non-bond}+F_{a}n\left(\theta \right)+\sqrt{2D_{t}}\Gamma_{a}\left(t\right)\\ \frac{d\theta }{dt}=\sqrt{2D_{r}}\Gamma_{\theta } \end{array}$$

where $m_{a}$ is the mass of the active particle and $\gamma_{a}$ is the friction coefficient subject to the active particle in pure solvent. $D_{t}$ and $D_{r}$ are the translational and rotational diffusion coefficients of active particles, respectively, satisfying the relation $D_{r}=3D_{t}/\sigma_{a}^{2}$, where $\sigma_{a}$ is the diameter of the active crowder. $F_{a}$ represents the amplitude of active force with orientation specified by the unit vector along the propelling axis of the particle, n(θ)=(cosθ,sinθ). The angular variable θ evolutes according to the second equation of (5), describing a simple rotational diffusion.

The solvent-induced Gaussian white-noise for both the translational and rotational motion are characterized by zero mean and the unit matrix of the correlational function, i.e., $\langle \Gamma_{a}\left(t\right)=0\rangle$, $\langle \Gamma_{a}\left(t\right)\Gamma_{a}\left(t^{\prime }\right)\rangle =I\delta \left(t-t^{\prime }\right)$, and $\langle \Gamma_{\theta }\left(t\right)=0\rangle$, $\langle \Gamma_{\theta }\left(t\right)\Gamma_{\theta }\left(t^{\prime }\right)\rangle =\delta \left(t-t^{\prime }\right)$.

It should be pointed out that this form of active force $F_{a}\left(\cos\theta ,\sin\theta \right)$ indicates a mean-field assumption that the active particle continuously releases energy at any given moment between two adjacent time steps of our simulation. This is a suitable model for a wide range of self-motile colloidal particles [42]. For instance, Howse et al. [43] studied the motion of an artificial microscale swimmer that used a chemical reaction catalyzed on its own surface to achieve autonomous propulsion. It was found that the trajectories of the active swimmer exhibited directed motion at short time scales. The motion reverted to a random walk over long time scales, where the orientation and direction of motion of the particle were randomized by its rotational diffusion. Obviously, in some cases, active forces are induced by slower diffusion-controlled processes, and the active force may become a random event other than a continuous force within a simulation time step. In such a situation, the assumption adopted here can be violated.

The bead mass and diameter of the probed polymer chain $m_{b}$ and $\sigma_{b}$ were chosen as the mass and length units. $k_{B}T$ was set to the energy unit. Then, time was scaled by $\tau =\sqrt{m_{b}\sigma_{b}^{2}/k_{B}T}$. In our simulation, we fixed the probed chain length *N* = 70. The non-bond interaction strength was $\epsilon =40k_{B}T$. For bond-interaction, the spring constant $k_{b}$ was set to be $2000k_{B}T/\sigma_{b}^{2}$. Densities of the active particle and polymer monomer were assumed to equal. Besides, for bond-angle interaction, the rigidity parameter κ was variable parameter in the latter simulation.

The velocity Verlet method was used to integrate Newton’s equation of motion. A Langevin thermostat was employed to maintain temperature with a low friction for an adequate sampling of the conformational space of the system. The friction coefficient $\gamma_{b}$ exerted on the polymer beads was set to be $1.0m_{b}\tau^{-1}$. The simulation integration time step Δt=0.0002τ. After initial equilibration of the system, the simulation was then run for ∼$3\times 10^{8}$ time steps, and data were obtained every 1000 steps. The entire simulation was repeated 20 times with different random choices of initial system conformations. Ensemble averages were obtained as a time average within each run, which was then averaged over different simulations to compute the quantities of interest.

The entire system was enclosed in a two-dimensional $L_{b}\times L_{b}$ box with $L_{b}=50\sigma_{b}$. According to our evaluation, the gyration radius of the probed polymer with the chain length was several times $\sigma_{b}$. The box width prescribed in our model was as large as about six-times the gyration radius of the probed chain, as suggested in some literature reports [44,45]. Therefore, the finite-size effect has been sufficiently avoided.

## 3. Results and Discussion

Now, we are able to reveal the stochastic conformations of a semi-flexible chain in an active bath based on the simulation model and method. The variable parameters included the rigidity κ of the polymer chain, active force $F_{a}$ and diameter $\sigma_{a}$ of the active particle, as well as the volume fraction ϕ. Through analyzing the gyration radius $R_{g}$ and its distribution, we can explore in detail the conformational change induced by both activity and crowding. The analysis for the size effect can be conveniently involved. Introducing different crowder sizes, the non-bond interaction energy between crowders and polymer beads will be modulated, through adjusting the diameter of the pairwise interacting particles. In addition, the translational and rotational diffusion coefficients $D_{t}$ and $D_{r}$ changed with $\sigma_{a}$ according to the simple relations described in the context following Equation (5). More importantly, varying the crowder size will also modify the dynamical persistence length of active crowders through changing $D_{r}$, and thus will lead to different activity effects.

**Figure 1:**
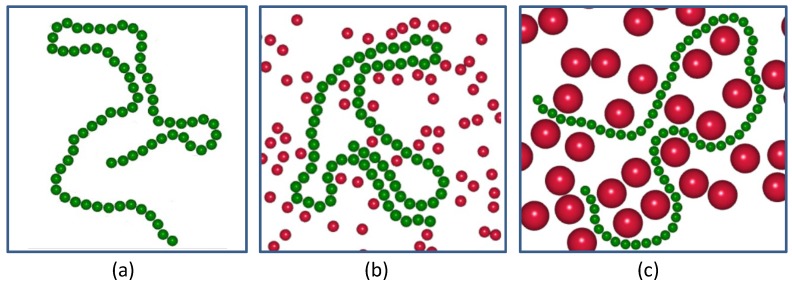
Snapshots of a probed semiflexible polymer with relatively small rigidity κ = 1.0 (green chain) suspended in pure solvent and (**a**) in active crowders (red particles) with a smaller size $\sigma_{a}$ = 1.0 (**b**) and a larger size $\sigma_{a}$ = 3.0 (**c**); at an equal volume fraction ϕ=0.1.

Figure 1 depicts the snapshots of the probed semi-flexible polymer with relatively small rigidity κ = 1.0 suspended in pure solvent (a), in small-sized active crowders, (b) and in large-sized active crowders (c), at an equal volume fraction.

In what follows, we will firstly investigate the activity-induced shrinkage or swelling of the probed chain under varying particle sizes, and then, we go further to elaborate the coupling effect between activity and crowding.

### 3.1. Activity Effect under Varying Crowder Sizes

As a key result, the scaled quantity $\lambda_{F_{a}}\equiv \langle R_{g}\rangle /\langle R_{g}\left(F_{a}=0\right)\rangle$ was introduced to quantify the activity-induced conformational change, where $\langle R_{g}\rangle$ and $\langle R_{g}\left(F_{a}=0\right)\rangle$ represent respectively the gyration radii of the chain in a bath with and without activity, and 〈〉 denotes the ensemble average. The volume fraction was fixed at ϕ=0.1. Polymers with different rigidities κ = 1.0, 5.0, 10.0, 20.0 were considered. Moreover, the active force ranged from 5–30, and three typical particle sizes $\sigma_{a}$ = 1.0, 2.0, 3.0 were specifically taken into account.

**Figure 2:**
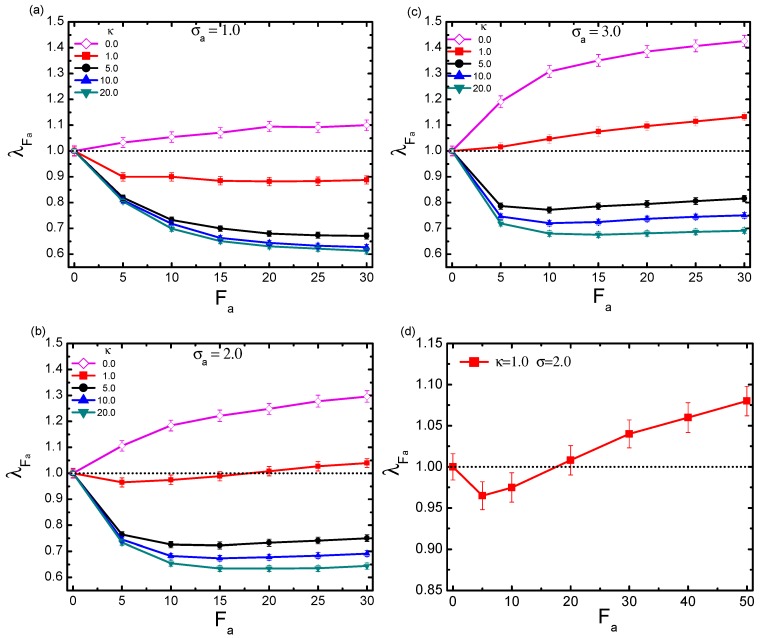
Scaled gyration radius $\lambda_{F_{a}}$ as a function of active force $F_{a}$ for polymers with different rigidities κ, under three typical crowder sizes: $\sigma_{a}$ = 1.0 (**a**); $\sigma_{a}$ = 2.0 (**b**); and $\sigma_{a}$ = 3.0 (**c**). (**d**) The magnification of the curve under κ=1.0 and $\sigma_{a}=2.0$, with a larger range of active force. The volume fraction is fixed at ϕ = 0.1. Error bars represent one standard deviation.

Figure 2 shows the dependence of $\lambda_{F_{a}}$ on the active force $F_{a}$ for polymers with certain rigidities (solid symbols), in the cases of $\sigma_{a}$ = 1.0, 2.0, and 3.0, respectively. For comparison, the fully-flexible chain characterized by κ = 0.0 is also evaluated (open symbols). Firstly, we notice that in the specific case of a fully-flexible chain, activity led to an constant swelling, which was consistent with the previous observations [36,37]. Moreover, such a swelling monotonically enhanced with the increment of both activity and crowder size. The underlying mechanism of activity-induced swelling for the flexible chain has been discussed in relevant literature. In brief, when a fully-flexible chain is immersed in an active bath, the active crowders are likely to intrude into the polymer chain and cluster in different regions. Consequently, an effective force will be generated, stretching the chain by pushing it in different directions. With increasing of the crowder size and active force, the distance moving along the orientation characterized by the dynamical persistence length becomes longer. As a result, the activity-induced effective force increases inevitably, leading to a more pronounced swelling on the probed chain, as we observed here. Very differently, the polymer with non-zero rigidity demonstrates much more complicated scenarios, showing possibilities of either shrinkage or swelling. The specific conformations not only depend on the polymer rigidity and the active force, but also the crowder size. Firstly, for polymers with relatively large rigidities κ≥5, it is clear that activity induces an evident collapse in the whole regime of $F_{a}$, for all cases of particle sizes. Harder et. al [37] performed similar numerical simulations for rigid polymers in a bath of active particles with a fixed diameter $\sigma_{a}$ = 1.0. Their novel finding was that collapse occurred at relatively small active forces $F_{a}<30$, resulting in a corresponding $\lambda_{F_{a}}≃0.6∼0.7$. Our simulation demonstrated a similar amplitude of collapse in the same force range in the case of $\sigma_{a}$ = 1.0 (a). In addition, they reported that the chain eventually reexpanded when the activity was larger $F_{a}>40$. Although such a large force calculation was missing in our simulation, similar reexpansion behaviors were reproduced at moderate forces in the cases of $\sigma_{a}$ = 2.0 (b) and 3.0 (c). Secondly, for a polymer with very small rigidity κ = 1.0, the conformational change behaved quite peculiarly. Under this situation, activity did not necessarily lead to a compacted conformation of the probed polymer. In fact, the polymer underwent a crossover from shrinkage to swelling with the increment of active crowder size. For κ = 1.0, in the bath of small active crowder size $\sigma_{a}$ = 1.0, the chain was compacted in the whole range of active force; while in the case of a large-sized $\sigma_{a}$ = 3.0, the chain was expanded on the contrary. In moderate crowder size bath $\sigma_{a}$ = 2.0, however, with increasing the strength of $F_{a}$, the chain firstly collapsed, then after a critical value of the active force, it followed the opposite swelling behavior. Such a shrinkage-to-swelling transition behavior is magnified in the inset of Figure 2b, with a larger range of active force. When a polymer with certain rigidity is immersed in an active bath, it undergoes the activity effect differently from the fully-flexible chain. On the one hand, for the probed polymer with a large rigidity, the active crowders have difficulty being intruded into the inner region of the chain. On the contrary, the crowders are inclined to accumulate in the outside region along the chain. The local asymmetries in the number of active crowders that cluster on either side of the rigid polymer can provide sufficiently large forces to bend it, resulting in an observable shrinkage behavior, as demonstrated. On the other hand, as the rigidity of the polymer is relatively small, which corresponds to a semi-flexible chain, the conformational change becomes rather delicate. This can be ascribed to the complicated entanglement effect between activity and rigidity. For a semi-flexible chain in a bath with small active crowders, the crowders have difficulty overcoming the chain rigidity to enter into the inner region of the chain. Instead, they cluster outside, generating bending forces and compacting the chain. As the crowders are bigger, they possess a longer dynamical persistence length and stronger ability to break the inhibition of the rigidity. The active crowders can realize an occupation of the inner region of the chain, exhibiting the role of stretching and swelling. The most interesting case surely occurs with a moderate crowder size. It is natural that the persistent moving ability of the active crowders and the impeding due to the chain rigidity become competitive in such a case. When active force is small, the rigidity effect is dominant; if active force is large, the activity effect prevails. Thus, this can be responsible for the crossover from shrinkage to swelling with the increasing of activity.

**Figure 3:**
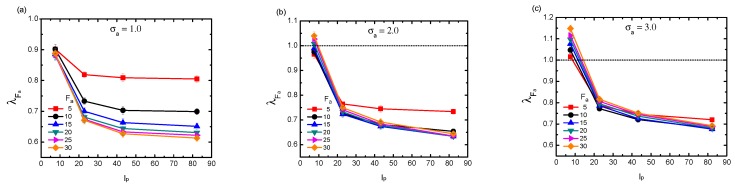
Dependence of scaled gyration radius $\lambda_{F_{a}}$ on the persistence length $l_{p}$ of the probed polymer with different active forces $F_{a}$, under three typical crowder sizes: $\sigma_{a}$ = 1.0 (**a**); $\sigma_{a}$ = 2.0 (**b**); and $\sigma_{a}$ = 3.0 (**c**). The volume fraction of the crowders is fixed at ϕ = 0.1. Error bars represent one standard deviation.

Figure 2 clearly demonstrates that $\lambda_{F_{a}}$ depends on not only active force $F_{a}$ and crowder size $\sigma_{a}$, but also on the rigidity κ. This parameter κ directly relates to another important characteristic length scale of the system, namely the persistence length (denoted as $l_{p}$) of the probed chain, which measures the contour distance over which the memory of the chain direction is lost. Careful analysis of the numerical data with respect to this length may provide a further intrinsic picture regarding the influence of chain rigidity. We evaluated $l_{p}$ according to the relation: $\langle \cos\theta \left(s\right)\rangle =e^{-s/l_{p}}$ where *s* denotes the distance between two segments along the chain and θ refers to the direction angle. According to our calculation, $l_{p}$ displayed a linear behavior proportional to κ (not presented). Figure 3 plots $\lambda_{F_{a}}$ as a function of $l_{p}$, for the three typical crowder sizes with varying active forces. As is shown, firstly, in the case of small crowder size σ=1.0 (a), the chain exhibited an ever-shrinkage behavior. The shrinkage degree monotonically increased with both $F_{a}$ and $l_{p}$. In the case of moderate crowder size $\sigma_{a}=2.0$ (b), the results became more complicated. As the chain was less rigid $l_{p}∼5.0$, it was firstly compacted under small $F_{a}$ and then underwent a gradual tendency to encounter swelling as $F_{a}$ increased. For the rigid polymer however, the chain was subject to an undoubted collapse. In the case of large crowder size σ=3.0 (c), when $l_{p}$ was small, the chain was mostly dominated by swelling behavior. With the increment of $l_{p}$, the chain turned to being compacted, with $\lambda_{F_{a}}$ here showing an evident convergence in bath activity.

**Figure 4:**
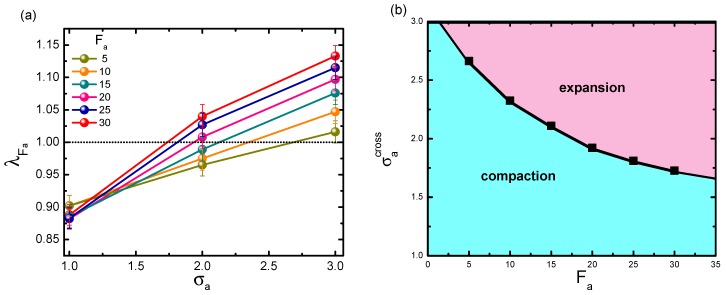
(**a**) Dependence of $\lambda_{F_{a}}$ on crowder size $\sigma_{a}$, under varying active forces $F_{a}$. Error bars correspond to one standard deviation. (**b**) The predicted phase diagram for the shrinkage-swelling transition, plotted in parameter space ($\sigma_{a},\;F_{a}$). In the calculation, the volume fraction is fixed at ϕ = 0.1, and the polymer rigidity is given as κ=1.0.

According to Figure 2, for a polymer with small rigidity, it is possible to observe a shrinkage-to-swelling transition, by modulating two relevant parameters, i.e., active force and crowder size. In order to specify the transition point in the $\sigma_{a}$-$F_{a}$ plane, $\lambda_{F_{a}}$ is plotted as a function of $\sigma_{a}$ at a prescribed $F_{a}$. Figure 4a displays the series curves under different active forces, with κ fixed to 1.0. Obviously, $\lambda_{F_{a}}$ exhibited a monotonic increment with respect to $\sigma_{a}$. The horizontal line separated the bottom collapse regime from the upper swelling regime. Though estimating the crossover point between this line and the $\lambda_{F_{a}}$ curve, we obtained the critical crowder size $\sigma_{a}^{cross}$ at each given active force. As crowder size exceeded the critical value under the corresponding $F_{a}$, the collapse-to-swelling transition appeared. Collecting the crossover points $\sigma_{a}^{cross}$ for varying active forces, we can plot the phase diagram for the transition between collapse and swelling in Figure 4b. The curve of $\sigma_{a}^{cross}$ as a function of $F_{a}$ exhibited a monotonic decay. As can be seen, in a small-sized bath, the probed chain mostly displayed the collapse state and had difficulty swelling, at least within the active force range in our simulation; while the chain easily swelled at very small $F_{a}$ in a larger-sized bath. The most interesting results happened in bath of moderate size. Whether the polymer was compacted or swelled was highly determined by the interplay between active force and active crowder size.

**Figure 5:**
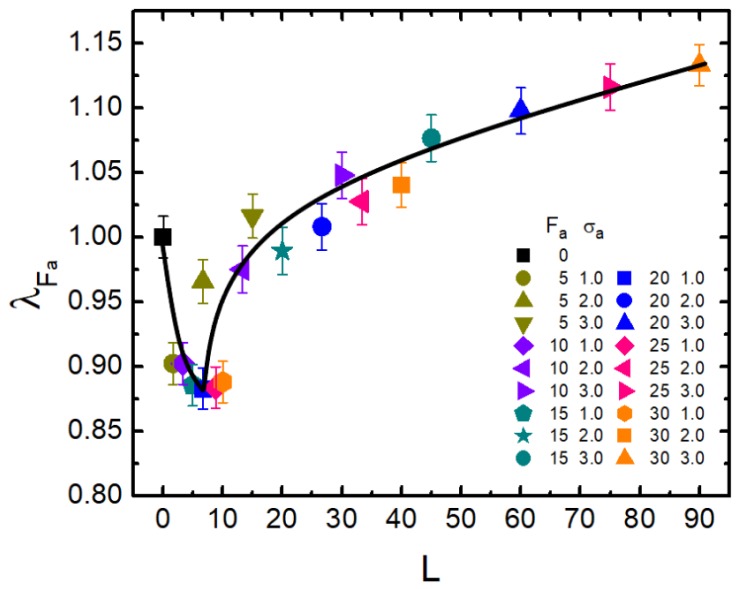
The scaled gyration radius $\lambda_{F_{a}}$ is plotted versus dynamical persistence length *L* for ϕ = 0.1 and κ=1.0. Error bars correspond to one standard deviation.

Furthermore, as demonstrated above, the dimension of a semi-flexible polymer can be effectively adjusted by active force $F_{a}$ and crowder size $\sigma_{a}$ of the bath. As mentioned in the Introduction, *L* characterizes the ability of the active particle to move along its original orientation. Thus, *L* should be a more proper parameter rather than $F_{a}$ to measure the extent of activity. It is therefore illuminating for us to investigate the relationship between $\lambda_{F_{a}}$ and *L*. Based on the results shown in Figure 2, we obtained Figure 5, which shows $\lambda_{F_{a}}$ as a function of the dynamical persistence length *L*. Very surprisingly, all data points apparently collapsed into a “universal” non-monotonic curve (solid line). In the small values of *L*, $\lambda_{F_{a}}$ followed a sharp decrease from 1.0–0.88 or so. After L>5, it oppositely gradually increased. $\lambda_{F_{a}}$ reached 1.13 when *L* was close to 90. The non-monotonic feature of $\lambda_{F_{a}}$ with respect to *L* also illuminated the contrasting role of activity, namely that activity promotes shrinkage in the small *L* region; while with the increasing of *L*, activity gradually played a greater role in expanding the polymer. In the large *L* domain, stronger activity led to more remarkable swelling.

Lastly, besides the scaled gyration radius $\lambda_{F_{a}}$ as depicted above, it is also interesting to analyze how activity and size effect play roles in modifying the polymer shape. Based on this starting point, the shape parameter *S* was examined, which is a characteristic quantity to measure the asymmetry in the shape of the probed chain conformation [46,47] and determined using the inertia matrix:

(6) $$T_{\alpha ,\beta }=\frac{1}{2N^{2}}\sum_{i,j=1}^{N}\left(r_{i\alpha }-r_{j\alpha }\right)\left(r_{i\beta }-r_{j\beta }\right)$$

where *N* is the total number of the probed chain beads. $r_{i\alpha }$ refers to the $\alpha^{\mathrm{th}}$ component of the position of bead *i*. For a two-dimensional description, α,β=x,y. The shape parameter *S* of the probed chain is defined as the ratio of two eigenvalues of the matrix *T* given by Equation (6), in the particular form of:

(7) $$S=\frac{\chi_{l}}{\chi_{s}}$$

where $\chi_{l}$ refers to the larger eigenvalue and $\chi_{s}$ is the smaller one.

**Figure 6:**
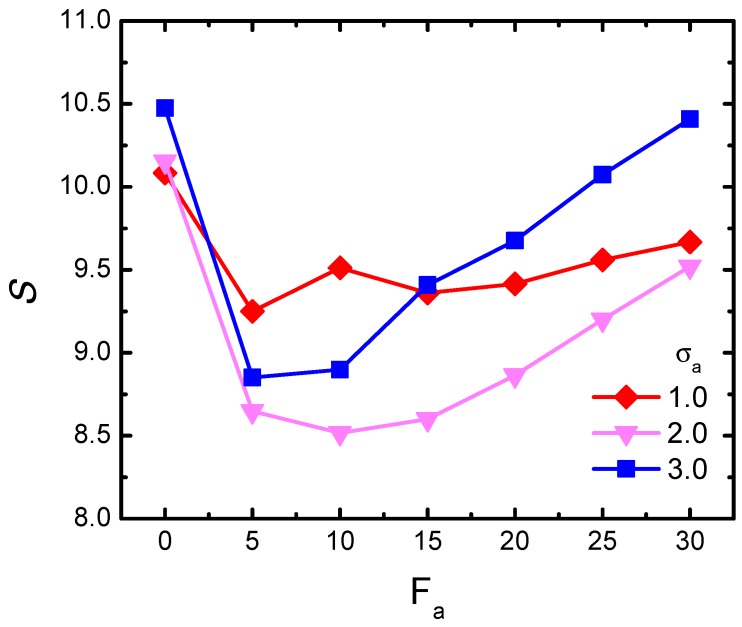
Shape parameter *S* of the semi-flexible chain as a function of active force $F_{a}$, for three different crowder sizes, given ϕ = 0.1 and κ=1.0.

We performed the calculation of *S* for the semi-flexible chain with κ=1.0. Figure 6 plots the shape parameter *S* with the variation of $F_{a}$ for three typical crowder sizes, respectively. As shown, firstly for the small-sized $\sigma_{a}$ = 1.0, *S* experienced only a slight decrease at lower forces and then gradually reached a plateau. This indicates that the activity had little influence on changing the polymer shape. However, as the crowder size increased (see $\sigma_{a}$ = 2.0 and 3.0), we observed a novel non-monotonic behavior of *S* with respect to $F_{a}$. At the beginning stage with small forces, *S* firstly decreased, indicating that the polymer had softened and became more flexible. With the increment of active force, *S* inversely increased. According to the physical implication of the shape parameter, a larger value of *S* indicated a more remarkable prolate contour, and thus corresponded to an effective extension of the gyration radius of the probed chain. The gradient of the increasing *S* in the case of $\sigma_{a}$ = 3.0 was obviously larger than that in $\sigma_{a}$ = 2.0. The sharp increasing in the larger-sized bath implies a heavier stretching effect, and definitely contributed to a stronger swelling phenomenon.

**Figure 7:**
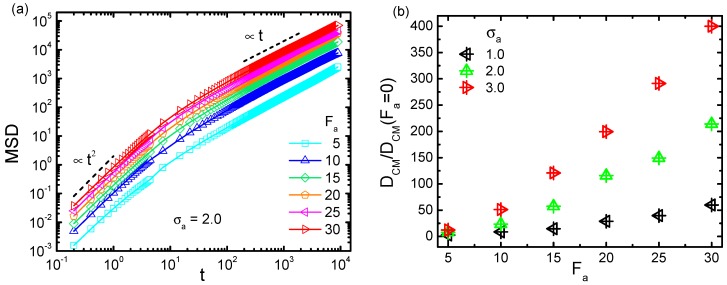
(**a**) Mean squared displacement (MSD) of the center of mass (CM) for the semiflexible chain with κ=1.0, in an active bath with $\sigma_{a}$ = 2.0 at ϕ=1.0. (**b**) The scaled diffusion coefficient $D_{CM}/D_{CM}\left(F_{a}=0\right)$ as a function of active force $F_{a}$, for $\sigma_{a}$ = 1.0, 2.0, and 3.0.

Besides the activity-induced conformational change of the probed chain as elaborated above, it is also desirable to investigate the activity effect on the polymer dynamics. According to previous studies [48,49], an active bath affects the dynamics of the immersed object as an effectively warmer temperature. In the present framework of Langevin simulation, the effective temperature was assumed to be maintained under each parameter setting. Such a temperature surely changed with varying the bath activity and crowder size. To investigate the associated heating effect, we analyzed the motional fluctuation of the probed chain. The mean squared displacement (MSD) of the center of mass (CM) of the chain was evaluated, based on which the long time diffusion coefficient $D_{CM}$ can also be estimated. Figure 7a displays the trajectories of MSD under different active forces $F_{a}$, for a semiflexible chain with κ=1.0, under ϕ=1.0 and $\sigma_{a}$ = 2.0. Obviously, the polymer diffused faster as $F_{a}$ became larger. The time dependence of MSD exhibited a typical superdiffusion in short time scales characterized by MSD(t) $∼t^{2}$, followed by a normal diffusion in the long time limit with MSD(t) ∼t. Figure 7b plots the dependence of $D_{CM}$ on the active force $F_{a}$, scaled by the value in the absence of activity $D_{CM}\left(F_{a}=0\right)$, for three crowder sizes $\sigma_{a}$ = 1.0, 2.0, and 3.0 respectively. As is shown, $D_{CM}/D_{CM}\left(F_{a}=0\right)$ monotonically increased with the increment of $F_{a}$. Meanwhile, the larger $\sigma_{a}$ was, the faster the probed polymer diffused. It can be concluded that the diffusion in an active and crowded bath was facilitated by increasing both active force and crowder size, indicating that the relevant effective temperature was enhanced correspondingly.

### 3.2. Activity-Crowding Coupling Effect

**Figure 8:**
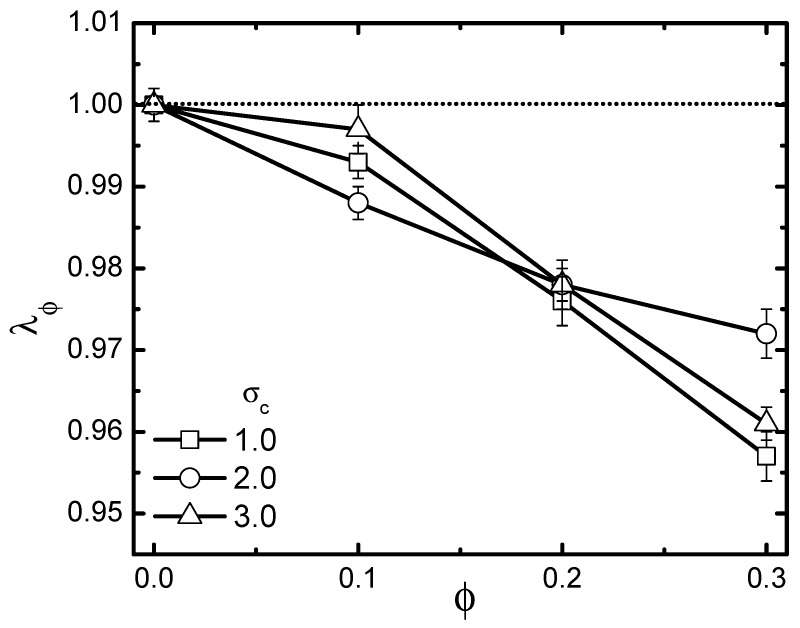
Dependence of scaled gyration radius $\lambda_{\phi }$ on volume fraction ϕ in a passive bath with three different crowder sizes. The polymer’s rigidity is given as κ=1.0. Error bars correspond to one standard deviation.

In the preceding subsection, we clarified the activity-size effect on the conformational change of the probed chain in an active bath. Evidently, the active bath that possesses a certain volume fraction is also a typical crowded environment. The probed chain was influenced by both activity and crowding. As is well known, the crowding effect is in general size-dependent. Thus, it is natural that activity and crowding effects will be inevitably coupled together, leading to very non-trivial shrinkage or swelling consequences for the probed chain. To emphasize such an issue, we analyzed the scaled gyration radius $\lambda_{\phi }\equiv \langle R_{g}\rangle /\langle R_{g}\left(\phi =0\right)\rangle$, with $\langle R_{g}\left(\phi =0\right)\rangle$ being the value of the gyration radius in a pure solvent without crowders. The volume fraction ϕ of crowders ranged from 0.1–0.3. The rigidity of the probed chain was fixed at 1.0. Firstly, the pure crowding effect through studying the specific case of a passive bath $F_{a}$ = 0 is investigated in Figure 8, which plots $\lambda_{\phi }$ as a function of ϕ under different crowder sizes. As is shown, crowding induced collapse, as expected. Moreover, $\lambda_{\phi }$ decreased monotonically with increasing ϕ, while exhibiting a non-monotonic dependence on the crowder size. Note that for a fully-flexible polymer in passive baths [39], previous studies revealed a monotonic enhancement of collapse as crowder size decreased. The non-monotonicity of the size-dependent collapse observed here for a semi-flexible polymer can be ascribed to be an interplay between the following two aspects. The first aspect is crowding-induced depletion. As is known, for a chain suspended in a crowded medium, there appears to be a depletion layer in the vicinity of the probed chain. Depletion originates from the fact that non-absorbing crowders tend to move away, avoiding a loss of configuration entropy [50]. An effective depletion force is generated, exerted on the pair of probed chain beads, which induces them to approach closer, resulting in chain collapse. It was revealed that depletion forces increase with decreasing crowder size. This is the reason why the flexible chain exhibited a monotonic enhancement of collapse as crowder size decreased. Besides depletion, for a semi-flexible chain, another important aspect should be taken into account. Not like a fully-flexible chain, which can realize a free interpenetration with the crowders, a semi-flexible chain will inevitably experience a spatial confinement due to the presence of crowders. As crowder size becomes larger, the confinement effect becomes more significant, leading to a more remarkable collapse of the chain. However, as mentioned above, the depletion was reduced in the case of larger crowders. It is clear that these two aspects are contrasting with the increment of crowder size, indicating that the size-dependent collapse can be very nontrivial, as demonstrated.

**Figure 9:**
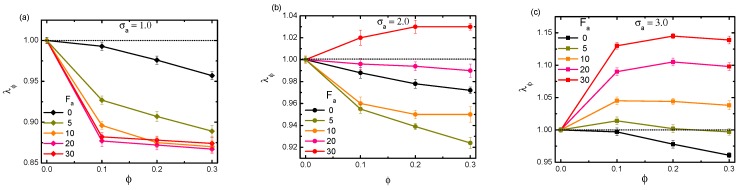
Dependence of scaled gyration radius $\lambda_{\phi }$ on volume fraction ϕ in an active bath, under varying active forces, with three different crowder sizes: $\sigma_{a}=1.0$ (**a**); $\sigma_{a}=2.0$ (**b**), and $\sigma_{a}=3.0$ (**c**). The polymer’s rigidity is given as κ=1.0. Error bars correspond to one standard deviation.

Then, activity was introduced into the system. Different from the pure crowding environment, the probed semi-flexible chain exhibited rich information of bath effects, in terms of volume fraction ϕ, active force $F_{a}$ and crowder size $\sigma_{a}$. Figure 9 portrays $\lambda_{\phi }$ as a function of volume fraction ϕ under varying active forces for $\sigma_{a}$ = 1.0 (a), 2.0 (b), and 3.0 (c), respectively. In the case of a small-sized $\sigma_{a}$ = 1.0, it shows that the probed chain was definitely compacted under varying active forces and volume fraction. Compared with the passive situation, activity greatly enhanced the degree of shrinkage, up to twice as large as that in a passive bath. According to the analysis in Section 3.1, in the case of $\sigma_{a}$ = 1.0, activity played a role in the shrinkage of the probed chain. Thus, it is easy to understand the similar collapse effects induced by both activity and crowding as superposed, responsible for the strengthened shrinkage behavior. With the increasing of crowder size, due to the complicated overlap of the size effect, the coupling between activity and crowding introduces quite an unexpected behavior. For moderately-sized $\sigma_{a}$ = 2.0, a very novel shrinkage-to-swelling transition was presented along with the increasing active force. Within this profile, as active forces were small $F_{a}$ = 5, 10, the probed chain exhibited a more compacted conformation than that in a passive bath. This is because small activity with moderate crowder size indicated a regime of shrinkage, depicted in Figure 4b. Therefore, activity positively intensified the crowding-induced collapse effect. As active force increased to a moderate value $F_{a}=20$, activity swelled the chain. The competition between the opposite effects of activity and crowding reduced the degree of shrinkage. While the active force was large enough $F_{a}=30$, swelling due to the presence of activity also increased to a certain degree, such that it overwhelmed the crowding-induced collapse effect, making the chain eventually display a swelled conformation. In the large crowder size case $\sigma_{a}$ = 3.0, the probed chain exhibited an evident swelling phenomenon for $F_{a}>5$, which indicated that the activity effect was a dominant factor. According to our study, as shown in Figure 4a, for an active bath with a large crowder size, activity was capable of inducing a remarkable swelling on the probed chain. For a certain large value of active force, the swelling effect by activity can easily exceed the collapse effect by crowding. The extent of swelling became more and more pronounced with the increment of active force.

**Figure 10:**
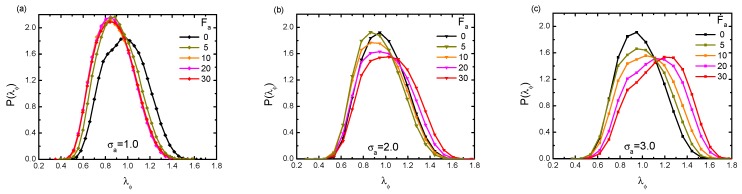
Profiles of distribution $P(\lambda_{\phi })$ under varying active forces, with three different crowder sizes: $\sigma_{a}=1.0$ (**a**); $\sigma_{a}=2.0$ (**b**); and $\sigma_{a}=3.0$ (**c**). The polymer’s rigidity is given as κ=1.0, and the volume fraction of the crowders is fixed at ϕ = 0.2.

Lastly, besides the average value of gyration radius, we went further to investigate the conformational information through analyzing the probability distribution of $R_{g}$. The distribution $P(\lambda_{\phi })$ is plotted in Figure 10, in terms of $\lambda_{\phi }$, under varying $F_{a}$ and crowder sizes $\sigma_{a}$. In the case of a small crowder size $\sigma_{a}$ = 1.0 (a), we observed that upon increasing the bath activity, $P(\lambda_{\phi })$ became sharper and clearly showed a gradual shift towards smaller values of $R_{g}$. The distribution curves converged to a universal one when $F_{a}>5$. Accordingly, this implies that the probed chain was able to reach conformations that were more collapsed compared to the one in the absence of activity. For moderately-sized $\sigma_{a}$ = 2.0 (b), the distribution first moved to smaller $R_{g}$ under $F_{a}$ = 5 and 10. Then, with increasing $F_{a}$, the distribution oppositely moved towards the right. This interesting behavior kept in accordance with the shrinkage-swelling transition phenomenon discussed above. In large size situation, $\sigma_{a}$ = 3.0 (c), with the increasing of active force, $P(\lambda_{\phi })$ showed a broader shape with a lower peak and kept moving towards the larger values of $R_{g}$. Evidently, such a broader shape of $P(\lambda_{\phi })$ clearly implies that due to activity, there was the coexistence of very different (collapsed or swelled) conformations.

## 4. Concluding Remarks

In the present work, we performed a systematic two-dimensional Langevin simulation study for the conformational change of a semi-flexible chain suspended in a crowded and active bath. Based on extensive analysis, the unusual behaviors of shrinkage or swelling of the probed chain have been investigated, showing an explicit dependence on polymer rigidity, active force, as well as crowder size. We specifically evaluated the two relevant functions about the gyration radius of the chain, $\lambda_{F_{a}}$ and $\lambda_{\phi }$, scaled by the values in the passive bath and in pure solvent, respectively. In addition, shape parameter *S* and the probability distribution $P(\lambda_{\phi })$ were also investigated to achieve a global picture to describe the conformational change. Particular attention has been paid to unraveling the important role of crowders’ size effect. Three typical crowder sizes were specifically taken into account, and a comparative study was carried out.

According to our analysis, the fully-flexible chain in an active bath will definitely swell, while rigid polymers suffer an opposite effect of activity-induced collapse. These results are consistent with the observations reported in the literature so far. While for a semi-flexible chain characterized by a small rigidity, as it was exposed to an active bath, we newly found out that the activity of the bath led to quite peculiar effects, which were demonstrate to be very discrepant from the results of the former two cases. The semi-flexible chain was compacted or expanded, explicitly depending on not only the active force $F_{a}$, but also the crowder size $\sigma_{a}$. A phase diagram in the parameter space of ($\sigma_{a},F_{a}$) was depicted to clarify the collapse and expansion regimes. It showed that in the case of a small-sized bath, activity mainly resulted in a collapsed conformation of the probed chain; while in the large-sized case, activity played the role of swelling. The most interesting situation occurred as the bath was packed with moderately-size particles, for which the shrinkage-to-swelling transition with the increasing of active force could be involved. Furthermore, from the viewpoint of the anomalous size effect, we examined the variation of the reduced gyration radius $\lambda_{F_{a}}$ in terms of the dynamical persistence length *L*. Surprisingly, we observed a well-scaled behavior of $\lambda_{F_{a}}$, showing a clear decrease-increase non-monotonic dependence on *L*. When *L* was small, increasing of activity led to a facilitated shrinkage. While in the large *L* region, activity started to expand the polymer. The consequent swelling effect became more pronounced when the dynamical persistence length of the active particle tended to become larger.

In addition, we elaborated the intriguing coupling effect between activity and crowding. Crowding of the bath induced an evident collapse of the probed chain. When crowding agents were active, activity and crowding we re inevitably entangled with each other and brought about a synchronous modulation to the chain conformation. Most importantly, both activity and crowding effects were crucially related to the crowder size. When crowder size was small, activity induced a compacted conformation of the chain, similar to the crowding effect. The coupling effect was positive, leading to a further strengthened shrinkage of the probed chain. In moderately-sized cases, with the increasing of activity, swelling due to activity started, which was opposite the crowding-induced collapse. Consequently, activity and crowding can be competitive, which is surely the reasonable origin of the non-trivial collapse-swelling transition, as demonstrated. In the large crowder size situation, activity provided a more remarkable swelling of the probed chain, such that activity possibly became a dominant factor, overwhelming the crowding effect. The chain therefore eventually experienced an apparent swelling behavior, as expected.

Note that all the above results were obtained at a fixed non-bond interaction strength ϵ=40.0 in the repulsive Weeks–Chandler–Andersen (WCA) potential given by Equation (3). From the viewpoint of conventional polymer-solvent theory, such a coefficient choice is somewhat critical, since it determines the “quality” of the solvent. In a passive bath, it is true that a greater value of ϵ would imply a worse solvent where the chain would collapse more easily; while a small value corresponds to a better solvent where the chain undergoes less collapse, and even would never collapse when this coefficient is small enough. Nevertheless, considering an active bath, besides the direct forces coming from non-bond interactions, activity induces clustering and trapping phenomena near the semi-flexible polymer boundary, and thus generates effective forces exerted on the chain. In the case of small activity, we expect different coefficients ϵ of the non-bond interaction to lead to significantly different results; while as activity reaches a certain extent, the variation of this coefficient would only bring about a small influence to the phenomenology.

Our study provides a basic understanding of how semi-flexible polymer conformations are affected in a two-dimensional (2D) active bath. The two-dimensional setup we used is realizable in experiments and relevant for biological systems. For instance, one may consider a DNA-like stiff chain in a two-dimensional *Bacillus subtilis* suspension [51,52]. Surely, more simulation is necessary to understand the three-dimensional (3D) case. The intriguing crowding-activity coupling effect observed in a 2D space is peculiar, which should be discrepant from that in a 3D confinement. In fact, the probability of a collision between active particles and the probed chain in 3D systems will be reduced; while the ability of the chain to effectively trap active particles in three dimensions is weakened. As a result, active particles in 3D bath would present different clustering and trapping phenomena, which generate discrepant effective forces on the chain. Even thought, we may expect that a similar shrinkage-swelling crossover might also occur in a 3D system, but following a different scenario regarding the size and activity effect on collapse or swelling. The comprehensive analysis of the conformational change of a semi-flexible chain embedded in a 3D bath of active particles is desirable, which is a subject worth studying in the future.

The study in the present paper provides a systematic analysis of the unusual collapse and swelling of a semi-flexible chain in an active bath. We hope that this work can provide a deeper insight into the activity and crowding effects on the conformational change and the relevant kinetics of biomolecules in more complicated active and crowded media.
